# The Reg protein family: potential new targets for the treatment of inflammatory bowel disease and colorectal cancer

**DOI:** 10.3389/fgstr.2024.1386069

**Published:** 2024-09-12

**Authors:** Anqi Yao, Cuilan Huang, Xuyang Wang, Renmin Zhou, Wujuan Hao, Qiong Lin

**Affiliations:** ^1^ Wuxi People’s Hospital Affiliated to Nanjing Medical University, Wuxi, Jiangsu, China; ^2^ Wuxi People’s Hospital Affiliated to Nanjing Medical University, Wuxi Children’s Hospital, Wuxi, China; ^3^ Digestive Department, Affiliated Children’s Hospital of Jiangnan University, Wuxi, China

**Keywords:** Reg protein family, inflammatory bowel disease, colorectal cancer, Crohn’s disease, ulcerative colitis, gastrointestinal microbiome

## Abstract

Inflammatory bowel disease (IBD) comprises Crohn’s disease (CD) and ulcerative colitis (UC), both characterized by chronic intestinal inflammation and an elevated risk of colorectal cancer due to persistent inflammation. The Regenerating gene (Reg) family proteins exhibit properties that promote cell proliferation, inhibit apoptosis, reduce inflammation, combat microbial infections, and potentially modulate the immune system. There is increasing evidence of the potential function of the Reg family of proteins in the development of IBD and colorectal cancer, but the exact mechanism of action of the Reg family of proteins has not yet been fully clarified. In this paper, we reviewed the Reg protein family’s involvement in the development of IBD by regulating intestinal microbes and immunity to maintain intestinal homeostasis. We also explored its possible regulatory mechanisms and signaling pathways in the progression and treatment of colorectal cancer, which is expected to serve as a target and a new biomarker for the treatment of IBD and colorectal cancer in the future.

## 1 Introduction

Inflammatory bowel disease (IBD), an idiopathic inflammatory disease of the intestines with clinical manifestations of diarrhea, abdominal pain, and even bloody stools, includes Crohn’s disease and ulcerative colitis and involves the ileum, rectum, and colon (1). In the 21st century, IBD has become a widespread ailment that is on the rise in both developed and developing countries. A regression analysis showed that the age-standardized prevalence of IBD in 2019 was 15.42% in Africa, 59.25% in Asia, and 147.82% in Europe, showing a significant upward trend, and the increasing global burden of IBD will pose a huge challenge to healthcare systems around the world (2, 3). IBD is a chronic progressive disease, with the majority of patients experiencing recurrent disease flares over the course of a long illness, reducing quality of life and increasing the incidence of psychological problems significantly (4). In addition, long-term chronic inflammation in the gut can induce DNA damage through oxidative stress, leading to the activation of pro-oncogenes and inactivation of oncogenes, which ultimately heightens the susceptibility of patients to develop colorectal cancer (5).

A complete understanding of the causes and development of IBD remains elusive, potentially arising from a complex interplay between factors including immune response, genetic predisposition, environmental influences, and microbiota (6). There is an increasing amount of evidence indicating that the disruption of the gut microbiota’s ecological balance serves as a catalyst for IBD (7). Disruption of the mucosal barrier caused by ecological dysregulation results in the persistence of inflammatory and carcinogenic effects. The presence of specific harmful bacteria, such as *Escherichia coli* and *enterotoxigenic Bacteroides fragilis*, can trigger the release of proinflammatory and oncogenic substances, thereby increasing the susceptibility to colorectal cancer among individuals with IBD (8). The gastrointestinal tract, being the body’s largest defense system, plays a vital role in defending against potential pathogens from entering the body; the dysfunction of the immune system within the intestines is also a significant contributing factor to the development of IBD (9).

The Reg protein family was first detected in pancreatic islet cells in 1988 (10). Since its identification, Reg has been proven to be associated with various health conditions including diabetes, inflammation of the gastrointestinal tract, and cancer (11). An increasing amount of evidence indicates that the expression of Reg is considerably elevated in the gastrointestinal tract of individuals with IBD, and it possesses antibacterial properties, anti-inflammatory effects, and tissue-healing capabilities (12). In addition, the expression of the Reg protein family has been observed in cancer and shown to impact prognosis. These proteins have potential applications as diagnostic markers or therapeutic targets for gastrointestinal tumors (13).

This paper examines the possible contribution of the Reg protein family to the advancement and evolution of inflammatory bowel disease and colorectal cancer, foreseeing their potential as therapeutic targets and prognostic biomarkers in future applications.

## 2 Reg protein

Reg genes were first detected in pancreatic islet cells in 1988 (10). In rodents, the Reg protein family includes Reg1, Reg2, Reg3α, Reg3β, Reg3γ, Reg3δ, and Reg4, whereas in humans, it includes Reg1α, Reg1β, Reg3α, Reg3γ, and Reg4 (14). The Reg1 gene and its protein product are derived from pancreatic alveolar cells (15). Recent research has revealed an increase in the expression of Reg1 within the inflamed epithelial lining of the colon, potentially associated with the rejuvenation of colon mucosal cells and exhibiting anti-apoptotic properties (16, 17). Reg1α was significantly upregulated in ulcerative colitis-associated colorectal cancer (18). The expression of the Reg2 gene is typically observed in pancreatic alveolar cells. In normal circumstances, it seems that the presence of Reg2 is not necessary for preserving pancreatic islet function and maintaining glucose balance. However, when confronted with aging or obesity induced by a high-fat diet, Reg2 plays a beneficial role in supporting the proliferation and functionality of pancreatic β cells. This ultimately aids in sustaining insulin secretion and promoting glucose homeostasis (19).

Reg3 is an antimicrobial peptide expressed mainly in Paneth cells of the small intestine, which protects against bacteria and inhibits bacterial translocation, and Reg3 facilitates the maintenance of metabolic homeostasis in a variety of tissues and organs, such as the liver, intestines, and skin (20). Reg4 was first identified in high-throughput sequencing analysis of inflammatory bowel disease libraries, and its expression is associated with infection and inflammation, with pro-proliferative and anti-apoptotic effects in a number of gastrointestinal tumors, such as colorectal, hepatocellular, and gastric cancers (21,) (22).. In summary, the Reg protein family exhibits a close relationship with inflammation and cancer in the gastrointestinal tract, necessitating a comprehensive understanding of their role in inflammatory diseases. Consequently, targeting Reg proteins could potentially pave the way for novel therapeutic interventions.

## 3 Role of Reg proteins in the pathogenesis of IBD

### 3.1 Maintenance of intestinal homeostasis by Reg proteins

Gut microbes act as a hub linking the external and intestinal environments and play roles such as pathogen defense and immune defense in the host. Gut microbes are important for maintaining human health and mediating disease. For patients with inflammatory bowel disease (IBD), there is an imbalance in the composition of intestinal microorganisms, characterized by a reduction in the abundance of Firmicutes and an elevation in Bacteroidetes and Actinobacteria (7). In addition, ecological dysregulation of the gut microbiota may induce intestinal inflammation by upregulating Enterobacteriaceae, increasing lipopolysaccharide (LPS) production, and activating inflammatory signaling pathways (23).

**Figure 1:**
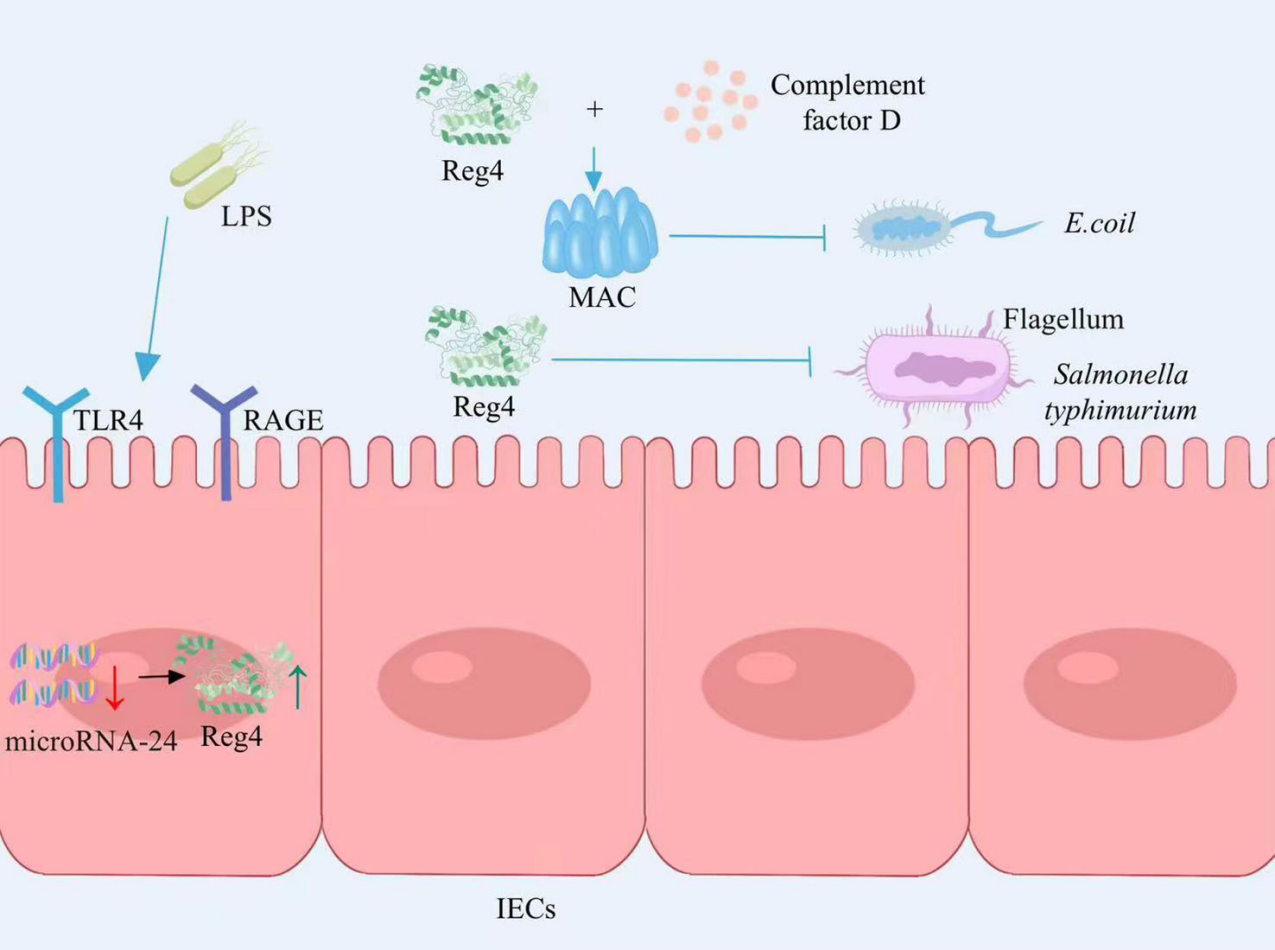
Reg4 maintains intestinal homeostasis. LPS signal transduction through the RAGE/TLR4 receptor mediated intracellular inhibition of microRNA-24 expression, thereby promoting the expression of Reg4, promoting the proliferation of intestinal epithelial cells. Reg4 and the complement factor D-mediated membrane attack complex inhibit *E. coli* colonization. Reg4 binds to the flagella of *S. typhimurium* and inhibits bacterial movement, thereby inhibiting bacterial colonization and reducing the host inflammatory response. LPS, lipopolysaccharide; RAGE, Receptor of Advanced Glycation Endproducts; TLR4, Toll-like receptor 4; IECs, intestinal epithelial cells; MAC, membrane attack complex; *E. coli*, *Escherichia coli*. The figure was created with Photoshop.

The expression of Reg4 is increased in the intestinal epithelium among individuals with IBD (24). LPS is a constituent of the external cellular barrier in Gram-negative bacteria. It can be mediated by RAGE/TLR4 receptors in intestinal epithelial cells, transferring LPS signals to cells and then promoting Reg4 gene expression by inducing microRNA-24 downregulation. Reg4 acts as a growth factor for intestinal epithelial cells to promote the proliferation of intestinal mucosal epithelial cells (25) (**Figure 1**). The study by Qi and colleagues found that Reg4 and complement factor D-mediated membrane attack complexes potentially contribute to the preservation of intestinal equilibrium through the eradication of inflammatory *Escherichia coli (* 26) (**Figure 1**). Recently, the expression of Reg4 was increased in the mouse model of *Salmonella typhimurium* infection. As a protein with antibacterial properties, Reg4 hinders the mobility of *Salmonella typhimurium* bacteria by selectively attaching to their flagella, which effectively impedes bacterial colonization and diminishes the host’s inflammatory reaction (27) (**Figure 1**). Therefore, Reg4 may be important for the future development of new drugs for infection-associated intestinal inflammation.

**Figure 2:**
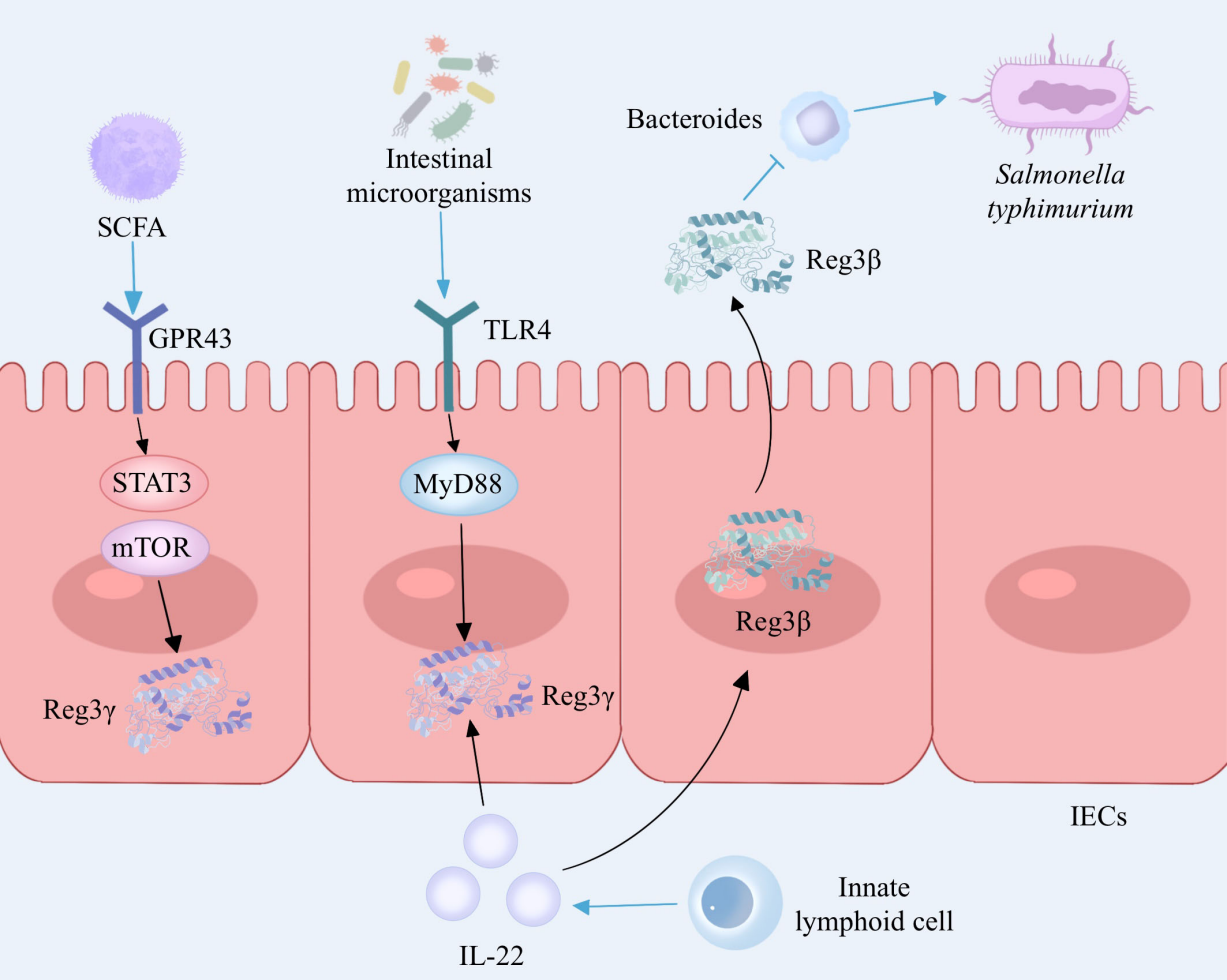
The interaction between Reg3 and gut microbiota. Intestinal microorganisms induce the secretion of Reg3γ by Paneth cells via the TLR4/MyD88 signaling pathway while also activating dendritic cells and promoting the proliferation of innate lymphoid cells. Innate lymphoid cells secrete IL-22 to enhance the production of Reg3γ in intestinal epithelial cells. Gut microbiota-derived short-chain fatty acids promote Reg3γ production in intestinal epithelial cells by activating mTOR and STAT3 via GPR43. IL-22 stimulated Paneth cells to produce Reg3β, which prolonged the intestinal colonization of *S. typhimurium* by inhibiting *Bacteroides*. TLR, Toll-like receptor; MYD88, myeloid differentiation factor 88; IL-22, interleukin-22; IECs, intestinal epithelial cells; SCFA, short-chain fatty acid; GRP43, G protein-coupled receptor 43; mTOR, mammalian target of rapamycin; STAT3, signal transducer and activator of transcription 3. The figure was created with Photoshop.

Reg3γ, an antimicrobial peptide belonging to the C-type lectin family, is synthesized and released abundantly by Paneth cells in the small intestine, which plays a crucial role in host defense by exhibiting potent bactericidal properties (28). Intestinal microorganisms can stimulate Paneth cells to secrete Reg3γ through the Toll-like receptor (TLR)/MyD88 pathway and can also stimulate the proliferation of innate lymphoid cells (ILCs) by activating dendritic cells (DCs). ILCs secrete IL-22 to induce the production of Reg3γ in intestinal epithelial cells, thereby inhibiting the colonization of Gram-positive bacteria and enhancing the ability of intestinal epithelial cells to inhibit bacterial survival (29,) (30). (**Figure 2**). Diet is closely related to inflammatory bowel disease, and a high-fat diet is considered to be a cause of IBD. The immune equilibrium in the intestine can be disturbed by a diet rich in fats, leading to potential harm to the integrity of the intestinal barrier, alterations in immune cells, and modifications in the composition of intestinal microbiota (31). The intestinal microorganisms exhibit distinct rhythms, and high-fat diets induce disease by modifying the microbial community in the ileum and disrupting the circadian rhythm of microorganisms. In a mouse model, it was observed that the intestinal microbial rhythm primarily relies on the dynamic interactions between diet, host, and microbes. The circadian pattern of Reg3γ expression is influenced by the presence of intestinal microbes. Reg3γ might contribute to preserving the ecological balance within the community and promoting intestinal health in hosts (32). Shin et al. (33) reinforced the presence of advantageous microorganisms in the gut, such as *bifidobacteria* and *lactobacilli*, by implementing measures like introducing inulin or conducting surgical procedures on the intestines. These microbial communities stimulated the expression of Reg3γ, leading to improved glucose tolerance and maintenance of intestinal homeostasis, thereby yielding metabolic advantages. In addition, the presence of probiotics resulted in an enhancement of intestinal mucosal barrier function in wild-type mice when compared to Reg3γ-knockout mice. This indicates that the improvement of intestinal barrier function and the connection between microbiota and host physiology necessitate some level of involvement from Reg3γ (33). Recently, a study on fecal microbiota transplantation revealed that the gut microbiome contributes to mediating diet-induced increases in ileal Reg3γ and Reg3β expression, as well as circulating levels of Reg3β (34). Additionally, gut microbiota-derived short-chain fatty acids can promote intestinal epithelial cell Reg3γ production and maintain intestinal homeostasis via G protein-coupled receptor 43 (GPR43) activation of mTOR and STAT3 (35) (**Figure 2**). In conclusion, Reg3γ, as part of the host immune system, can maintain intestinal homeostasis by inhibiting bacterial colonization and mediating gut microbiota–host interactions.

Reg3β and Reg3γ exhibit structural similarities, share comparable expression patterns and regulatory mechanisms, and demonstrate antimicrobial properties and the ability to promote tissue healing (36). Recent research has shed light on the crucial function of IL-22 in controlling inflammation within the intestines and preserving the integrity of the epithelial barrier. IL-22 has the ability to induce Paneth cells to secrete Reg3β, which serves as an effector downstream of IL-22 with potent antibacterial properties (37) (**Figure 2**). In addition, Shindo and colleagues found elevated expression of IL-6 and chitinase-like 3, a marker of tissue-repairing macrophages, in the colons of Reg3β knockout mice compared to wild-type mice after the administration of DSS, which may be associated with worsening of colitis, suggesting that Reg3β contributes to the alleviation of colitis in mice (38). Therefore, it is speculated that Reg3β may function as an antimicrobial protein and exert protective effects on IBD by inhibiting the colonization of pathogenic bacteria. These studies indicate that Reg3β contributes to antimicrobial activity, anti-inflammatory response, and tissue regeneration, thereby offering a novel potential therapeutic target for intestinal disorders.

However, dysregulation of antimicrobial peptide production may disrupt gut flora homeostasis and may be associated with the development of IBD. Jang et al. (39) found that high levels of antimicrobial peptide Reg3 in the intestines of IBD patients could lead to *Enterococcus faecalis* depletion, while *Enterococcus faecalis* could increase the secretion of IL-22 to resist intestinal injury. Reg3β promotes sustained intestinal colonization and diarrhea duration of *Salmonella typhimurium* by inhibiting *Bacteroides* in a murine model of *Salmonella* diarrhea (40) (**Figure 2**).

These results suggest that although the Reg protein serves a crucial function as an antimicrobial peptide in defending against pathogenic microbial infections and maintaining intestinal homeostasis, its overactivation may disrupt the symbiotic relationship between the host and the intestinal microbiota and induce intestinal diseases. In the future, we can further explore the interaction between Reg protein–bacteria–IBD and elucidate their regulatory mechanism, which is of great significance for intestinal health promotion and the prevention and treatment of IBD.

### 3.2 Reg proteins regulate intestinal immunity

Immune cells in the gut, including macrophages, dendritic cells, and intrinsic lymphocytes, recognize invading pathogenic microorganisms and kill and eliminate them. In addition, various subpopulations of T and B cells, such as helper cells (Th1, Th2, Th17, and Th9), also play a major part in IBD.

Both regulatory T cells (Tregs) and Th17 are derived from CD4^+^ T cells. Transforming growth factor-β (TGF-β) plays a crucial role in modulating the adaptive immune response (41). TGF-β stimulates primitive CD4^+^ T cells and induces the development of Th17 cells and Treg cells. Treg cells play a negative role in immune regulation, secreting cytokines with anti-inflammatory properties, such as IL-4 and IL-10, mainly by inhibiting the activity of Th cells and reducing the production of proinflammatory factors, therefore controlling inflammation, which is an important regulator of intestinal homeostasis (42). Under normal conditions, Th17 cells act as a defense mechanism against the onset of infections and maintain intestinal immune homeostasis, whereas in immune dysregulation, overproliferation of Th17 cells induces an abnormal immune response in the body and mediates the development of immune-related diseases, such as IBD (43). Th17 and Treg cells are linked to each other in differentiation through TGF-β and inhibit each other in function. Together, they maintain the balance of the body’s immune microenvironment and, if disrupted, can lead to the development of many autoimmune diseases, including IBD (44).

In its quiescent state, TGF-β exists in a transcriptionally repressed conformation and necessitates activation to manifest its biological effects (45). Integrin αvβ8 on DCs was found to activate TGF-β and thus promote Th17 development by mouse models, and defects in Th17 induced by DCs lacking integrin αvβ8 could be rescued by the addition of exogenous TGF-β (46, 47).

Recent studies have found that the deficiency of Regγ promotes the upregulation of integrin αvβ8 on DCs, thereby inducing the activation of TGF-β maturation and facilitating the differentiation of Th17 cells (48). These findings suggest that Regγ could be a promising candidate for immunotherapy in IBD, making it a potential target for future research.

The etiology of IBD remains unclear; however, an abnormal response of the immune system leads to the development of IBD. Some members of the Reg family of proteins have been found to be autoantigens. RegII, regeneratively expressed in salivary gland tubular epithelial cells of SS patients, is involved in the autoimmune disease desiccation syndrome, which may affect the regeneration and function of the salivary glands (49, 50). Gurr et al. (51) found that RegII is a novel β-cell-derived autoantigen in NOD mice and that an autoimmune response against this protein may transform the regenerative process into an islet-destructive one, accelerating the development of type 1 diabetes. It is speculated that if Reg proteins are incorrectly recognized by the immune system as foreign antigens in IBD, they may also trigger or exacerbate an inflammatory response. Future studies need to further explore the detailed mechanisms of Reg proteins in IBD immunomodulation in order to better understand their roles in disease progression and provide new ideas for the diagnosis and treatment of IBD.

## 4 Role of Reg proteins in the development and therapeutic prognosis of colorectal cancer

### 4.1 Reg1α

Chronic inflammation of the gut in patients with IBD increases the risk of colorectal cancer, and patients with IBD accompanied by a long course and a wide range of lesions are at a considerably higher likelihood of developing colorectal cancer (52). A systematic review and meta-analysis revealed that the incidence of colorectal cancer in individuals diagnosed with UC was found to be 0.02% (95% CI 0.00–0.04) after a decade, 4.81% (3.26–6.36) after two decades, and 13.91% (7.09–20.72) after three decades (53). During the period of 1969–2017, a cohort study conducted in Sweden and Denmark, involving patients with IBD, revealed that individuals with Crohn’s disease had a 40% higher likelihood of developing concurrent colorectal cancer (CRC) compared to the general population (54). The pathogenic factors contributing to the development of colorectal cancer associated with inflammatory bowel disease encompass the extent and duration of chronic inflammation, genetic susceptibility, and the influence of symbiotic microbiota. Chronic inflammation induces oxidative stress-mediated DNA damage, thereby triggering oncogene activation and tumor suppressor gene inactivation (55).

**Figure 3:**
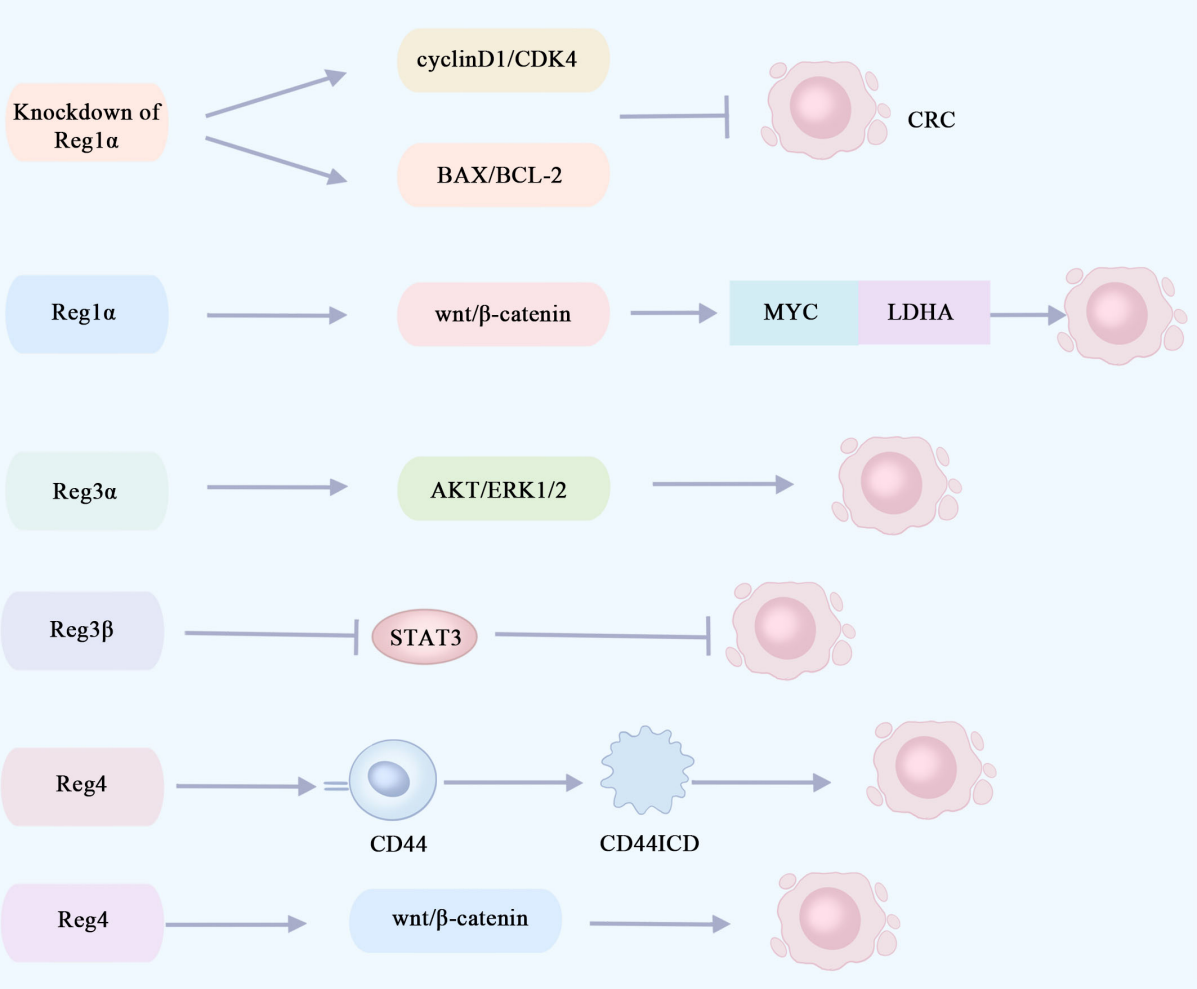
Reg proteins promote or inhibit signaling pathways involved in the proliferation of colorectal cancer cells. The knockout of Reg1α gene significantly impeded the viability of colorectal cancer cells and induced apoptosis via modulation of the cyclin D1/CDK4 pathway and the BAX/BCL-2 pathway. Reg1α enhances the expression of the MYC gene in colorectal cancer by activating the Wnt/β-catenin signaling pathway, thereby promoting aerobic glycolysis in cancer cells through its interaction with LDHA. This metabolic alteration contributes to the proliferation and metastasis of colorectal cancer cells. The activation of the AKT and ERK1/2 pathways by Reg3α may contribute to the promotion of colorectal tumorigenesis. The inhibition of STAT3 by Reg3β can effectively impede the initiation and progression of colorectal cancer tumors. Reg4 promotes colorectal cancer cell proliferation and stem cell formation by interacting with the CD44 receptor and inducing its proteolysis, thereby increasing the expression of the CD44 intracytoplasmic domain (CD44ICD). Reg4 promotes colorectal cancer stem cell formation through the Wnt/β-catenin pathway. CRC, colorectal cancer; CD44ICD, CD44 intracytoplasmic domain; CDK4, **cyclin-depend**ent kinases4; LDHA, lactate dehydrogenase A.

High expression of serum Reg1α in cancer patients was identified as an independent risk factor for cancer from an observational and cross-sectional study of 130 patients (56). Defective transduction of the p53 signaling pathway is an early event in the progression of developmental abnormalities to cancer. p53 immunohistochemistry can be used for the diagnosis of UC-associated colon cancer, and early mutations in P53 are detected in the intestinal mucosa of colorectal cancer patients (55, 57). Immunohistochemical analysis of colectomy samples from 31 patients with long-term UC revealed that Reg1α was significantly elevated in ulcerative colitis-associated tumor tissues and was significantly and positively correlated with p53 expression (58). Knockout of the Reg1α gene significantly suppresses the viability of colorectal cancer cells and then induces apoptosis via modulation of the cyclin D1/CDK4 pathway and the BAX/BCL-2 pathway (59) (**Figure 3**). The pro-proliferative and anti-apoptotic properties of Reg1α may contribute to the pathogenesis of colorectal cancer associated with inflammatory bowel disease. Reg1α may serve as a new biomarker for the diagnosis of colorectal cancer patients.

The primary energy source utilized by tumor cells is glycolysis, with aerobic glycolysis being recognized as an indicator of colorectal cancer (60). A recent study revealed that Reg1α enhances the expression of the oncogene MYC, which encodes a nucleoprotein, in colorectal cancer by activating the Wnt/β-catenin signaling pathway. Additionally, MYC forms a complex with lactate dehydrogenase A (LDHA) to promote aerobic glycolysis in cancer cells, thereby facilitating the proliferation and metastasis of colorectal cancer cells. Furthermore, elevated levels of Reg1α are indicative of an unfavorable prognosis for patients with colorectal cancer (61) (**Figure 3**). In the future, targeting the Reg1α/β-catenin/MYC/LDHA pathway could be a viable therapy option for individuals with colorectal cancer.

In addition to its involvement in the development of colorectal cancer, Reg1α also contributes to the treatment of this malignancy. The expression of Reg1α is upregulated in colorectal cancer cell lines, and genetic knockout of Reg1α has been shown to augment the sensitivity of these cells toward 5-fluorouracil (5-FU) chemotherapy (59).

### 4.2 Reg3α

Reg3α is not only a promising tumor marker but also plays a key role in the development of gastrointestinal malignancies. Previous research indicates that Reg3α can promote cell proliferation and inhibit apoptosis, including regulating keratinocyte differentiation and proliferation in damaged skin tissues (62, 63). Reg3α also functions as a growth factor that acts locally in hepatocytes, stimulating the proliferation and survival of these cells (64). In pancreatic cancer cells, Reg3a accelerates cell cycle progression by promoting the expression of cyclin D1 and enhances the expression of the anti-apoptotic gene Bcl2, and in pancreatic cancer cell lines SW1990 or BxPC-3, Reg3α promotes pancreatic cancer cell growth, proliferation, and tumor formation (65, 66). Chen et al. (67) observed a significant upregulation of Reg3α mRNA levels in gastric cancer tissues, which subsequently facilitated the proliferation and migration of gastric cancer cells via activation of the JAK2/STAT3 signaling pathway. Therefore, based on the intrinsic pro-proliferative and anti-apoptotic activities of Reg3α, it can be speculated that overexpression of Reg3α in colorectal cancer can promote colorectal tumor growth.

Earlier research has indicated that, in the presence of inflammation, Reg3α facilitates the proliferation of pancreatic cancer cells by activating the IL-6–JAK2/STAT3 signaling pathway (65). Elevated levels of IL-6 and sIL-6R are observed in the circulation and intestine of IBD patients, promoting colon cancer cell proliferation and tumor growth (68). Reg3α has been identified as a potential biomarker for the early detection of colorectal cancer, and Reg3α gene expression is upregulated in colorectal cancer tissues (69). Based on these findings, it is postulated that Reg3α may facilitate the initiation and progression of colorectal cancer in patients with IBD via activation of the IL-6–JAK2/STAT3 signaling pathway. In addition, Reg3α may promote colorectal tumorigenesis through activation of the AKT and ERK1/2 pathways, and the higher the expression level of Reg3α, the larger the colorectal tumor and the poorer the prognosis (70) (**Figure 3**). The growth of colorectal cancer cells, LOVO and RKO, was markedly suppressed upon the downregulation of Reg3α (70). Reg3α promotes cancer cell cycle progression and tumorigenicity by forming RNA–DNA triple-stranded bodies with lncRNA Reg1CP and is associated with poor patient prognosis (71). Overall, Reg3α may regulate the proliferation and apoptosis of colorectal cancer cells through multiple signaling pathways. It is considered a potential pathogenic gene for colorectal cancer and is expected to be a target for treating this disease.

However, Reg3α may also exert its function as a novel tumor suppressor. In a study conducted on mice, it was observed that overexpression of Reg3α led to the activation of T-cell-mediated immune response, resulting in a suppressive effect on colon adenocarcinoma. Additionally, analysis of clinical data revealed a positive correlation between higher levels of Reg3α expression and improved prognosis among patients diagnosed with colorectal cancer (72). The examination of human gastric cancer cell culture revealed that the promotion of tumor suppressor gene expression by Reg3α could potentially impede the proliferation of gastric cancer cells (73). Such inconsistent results of Reg3α may be due to different research objects or may be caused by differences in the selected pathological types of cancer cells, which need further research and exploration in the future.

To conclude, the role of Reg3α in cancer conditions is contradictory: in some studies, it can promote the proliferation of cancer cells, but some studies have also found that Reg3α may be a new type of tumor suppressor factor. Future research on Reg3α may be a new idea for cancer treatment.

### 4.3 Reg3β

Reg3β, a lectin, has also recently been found to be involved in the development of colorectal cancer. STAT3 is an important immunomodulatory factor, which plays a significant pathogenic part in colorectal cancer development, progression, and metastasis (74). In the mouse model of colorectal cancer, it was found that Reg3β could prevent colorectal cancer tumorigenesis and growth through its inhibitory effect on STAT3, and the expression of Reg3β was negatively related to the prognosis of colorectal cancer (75) (**Figure 3**). Increasing Reg3β expression in colorectal cancer could be a promising therapeutic strategy.

### 4.4 Reg4

Reg4 is highly upregulated in gastrointestinal malignancy. REG4 expression was significantly associated with a poorer overall survival rate and recurrence-free survival rate according to the amount of substrate (76). Li et al. (77) conducted immunohistochemical studies on colorectal cancer tissues, adjacent tissues, non-adjacent tissues, and adenoma tissues. They observed an upregulation of Reg4 expression in adjacent and adenoma tissues, while a decrease was noted in colorectal cancer tissues. These findings suggest that Reg4 overexpression may be an early event in colorectal carcinogenesis. Additionally, CD44 is recognized as a marker for tumor stem cells with its intracytoplasmic domain (CD44ICD) playing an essential part in cancer cell migration and proliferation (78). Bishnupuri et al. (79) (**Figure 3**) discovered that through its interaction with the CD44 receptor and subsequent induction of proteolysis, Reg4 can enhance the expression of the intracytoplasmic domain of CD44 (CD44ICD), thereby activating transcription of type D cyclin involved in regulating cancer cell proliferation. This ultimately promotes both colorectal cancer cell proliferation and stem cell formation. The correlation observed between Reg4 and CD44 or CD44ICD suggests that Reg4 may contribute to the enhancement of colorectal cancer cell proliferation and stem cell generation (80). KRAS is a common mutant oncogene in colorectal cancer (81). Hwang et al. (82) (**Figure 3**) found that mutant KRAS-induced Reg4 promotes colorectal cancer stem cell formation via the Wnt/β-catenin pathway. Recently, an engineered immunoglobulin (scFv-Reg4) was generated which can bind specifically to Reg4 and block its biological activity, significantly inhibiting cancer cell proliferation (83). These findings indicate that Reg4 could potentially play a role in the initiation and progression of colorectal tumors. In the future, it is anticipated that Reg4 may serve as a valuable biomarker for predicting the prognosis of colorectal cancer. Additionally, targeting Reg4 at the molecular level holds promise for gene therapy approaches in treating colorectal cancer.

5-FU is a chemotherapy drug commonly used in the treatment of colorectal cancer; however, resistance to 5-FU chemotherapy results in the failure of colorectal cancer treatment. Previous research has demonstrated that lipid droplet accumulation contributes to chemoresistance in colorectal cancer cells (84). Zhang and his team recently discovered that Reg4 enhances chemoresistance in colorectal cancer by suppressing the transcription of ACC1 or ACLY, thereby impacting lipid droplet synthesis and assembly (85). The scFv-Reg4 significantly enhances the apoptotic effect of 5-FU, thereby indicating its potential as a promising supplement for the treatment of gastrointestinal tumors (83). The above shows that knockdown of the Reg4 gene can enhance the sensitivity of colorectal cancer to chemotherapeutic drugs, suggesting that the prognosis of colorectal cancer patients can be improved by gene knockdown.

The expression of Reg4 is downregulated in chemoradiotherapy-sensitive colorectal cancer cells (86). It suggests that Reg4 may be a potential biomarker of sensitivity to radiotherapy in colorectal cancer and could help predict treatment response in patients undergoing RCT, thus enabling effective personalized treatment.

In brief, the involvement of the Reg protein in gastrointestinal malignant tumors is significant, suggesting its potential as a valuable biomarker for tumor diagnosis, prognosis, and targeted therapy. In forthcoming research, targeting the Reg protein could be explored to impede tumor growth, hinder proliferation, and overcome drug resistance.

## 5 Conclusion

The pathogenesis of inflammatory bowel disease remains elusive, involving the intricate interplay of environmental, genetic, immune, microbial, and other factors. Dysbiosis of the intestinal microbiota disrupts intestinal homeostasis and facilitates the onset and progression of IBD. Currently, clinical management primarily relies on aminosalicylates and immunosuppressants. However, prolonged medication usage is associated with heightened adverse reactions and imposes substantial time and economic burdens on patients. In addition, chronic inflammation of the intestinal tract increases the risk of colorectal cancer, so new targets need to be found to improve patient care. The Reg protein family is involved in the regulation of intestinal flora and plays an important role in the development and treatment of inflammatory bowel disease and colorectal cancer, and in the future, through an in-depth study of the relationship between Reg proteins and gastrointestinal inflammation and malignant tumors, we can further explore the signaling pathway that regulates the expression of Reg proteins to develop relevant drugs to achieve therapeutic goals.
